# Job vacancies at the European Centre for Disease Prevention and Control (ECDC)

**DOI:** 10.2807/1560-7917.ES.2025.30.50.202512186

**Published:** 2025-12-18

**Authors:** Eurosurveillance editorial team

**Affiliations:** 1 European Centre for Disease Prevention and Control (ECDC), Stockholm, Sweden

The European Centre for Disease Prevention and Control (ECDC) invites applications for the following positions:

**Principal Expert General Surveillance/Group Leader Surveillance Systems**
The application deadline expires on 23 January 2026.
**Scientific Officer Food-, Water-borne and Zoonotic Diseases**
The application deadline expires on 27 January 2026.
**Principal Expert Vector-borne and Zoonotic Diseases/ Group Leader Scientific Advice and Outbreak Response**
The application deadline expires on 30 January 2026.

For more information, please visit the ECDC recruitment platform: https://erecruitment.ecdc.europa.eu/?page=advertisement

